# The effects of early childhood probiotic intake on the association between prenatal micronutrient supplementation and neurobehavioral development in preschool children: a four-way decomposition analysis

**DOI:** 10.3389/fnut.2025.1614820

**Published:** 2025-05-21

**Authors:** Liwen Ding, Maolin Zhang, Esben Strodl, Xiaona Yin, Guomin Wen, Dengli Sun, Danxia Xian, Yafen Zhao, Yuxing Zheng, Feitong Liu, Ruibiao Hu, Lingling Zhao, Weikang Yang, Weiqing Chen

**Affiliations:** ^1^Department of Epidemiology and Health Statistics, School of Public Health, Sun Yat-sen University, Guangzhou, China; ^2^School of Psychology and Counselling, Queensland University of Technology, Brisbane, QLD, Australia; ^3^Maternal and Child Healthcare Hospital of Longhua District, Shenzhen, China; ^4^Biostime (Guangzhou) Health Products Ltd., Guangzhou, China; ^5^School of Health Management, Xinhua College of Guangzhou, Guangzhou, China

**Keywords:** neurobehavioral development, micronutrient supplementation, probiotic intake, preschool children, four-way decomposition, counterfactual attribution

## Abstract

**Background:**

Neurobehavioral developmental disorder (NDD) significantly impact children’s long-term wellbeing and contribute to global disease burden. While prenatal micronutrient supplementation has shown promise in improving fetal neurodevelopment, its association with offspring’s neurobehavioral outcomes remains controversial, and the potential effect of early childhood probiotic intake on this association is still underexplored. This study aimed to evaluate the association between prenatal micronutrient supplementation and neurobehavioral development in preschool children, and to explore and quantify the effect of early childhood probiotic intake on this association.

**Methods:**

We included 15,636 mother–child dyads in Shenzhen, China, in 2022. Mothers provided information on prenatal micronutrient supplementation (calcium, folic acid, iron, and multivitamins) and early childhood probiotic intake through a structured questionnaire. Neurobehavioral development was assessed using the Ages and Stages Questionnaire (ASQ-3). Logistic regression was used to examine the association between prenatal micronutrient supplementation and NDD across crude, adjusted, and full-inclusion models. The effect of early childhood probiotic intake on the association between prenatal micronutrient supplementation and NDD was evaluated through four-way decomposition analysis and quantified using counterfactual attribution under three scenarios.

**Results:**

Among the participants, 11.7% were identified with NDD. Prenatal multivitamin supplementation was significantly associated with a reduced risk of NDD (OR = 0.73, 95% CI = 0.66–0.81). Early childhood probiotic intake was associated with an enhanced protective effect (Total EOR = −0.33, 95% CI = −0.54 to −0.12), with 48% of the effect attributable to interactions. Early childhood probiotic intake could prevent an additional 73 NDD cases (a 59% increase), particularly benefiting the gross motor, fine motor and personal-social domains.

**Conclusion:**

Prenatal multivitamin supplementation has a protective effect against NDD in preschool children, and early childhood probiotic intake is associated with an enhancement of this protective effect. These findings underscore the potential effect of early-life dietary supplements for NDD prevention. Further studies are recommended to confirm these effects and explore underlying mechanisms.

## 1 Introduction

Neurobehavioral development refers to the development of the brain and nervous system in behavioral, cognitive, and emotional regulation, playing a crucial role in children’s academic success, mental wellbeing, professional prospects, and overall quality of life (1). Despite its importance, neurobehavioral development disorders remain prevalent worldwide, characterized by key functions disorders like perception, motor skills, and language (2, 3). The 2019 World Health Organization (WHO) report estimated that approximately 58 million children (7%) globally experienced developmental disorders, with neurobehavioral disorders accounting for more than half of these cases (4). In China, the reported prevalence of such disorders ranges from 3.2 to 13.9%, with the personal-social domain being the most affected (5–8). Severe cases may manifest as conditions such as autism spectrum disorder (ASD) and attention deficit hyperactivity disorder (ADHD), both of which are increasing in prevalence (9–11). These conditions impose significant challenges on affected individuals, their families, and society. For example, ASD is responsible for more than 691.5 disability-adjusted life years (DALYs) per 100,000 individual globally, ranking it among the top 10 neurological disorders (12). Families of children with ASD face approximately $3,020 in additional annual healthcare costs and substantial losses in parental productivity (13). The annual social cost for all ASD patients may reach $41.8 billion, accounting for approximately 3.76% of China’s total healthcare expenditure in 2020 (14). Therefore, early identification of influencing factors is essential to prevent severe neurobehavioral developmental disorders and mitigate long-term socioeconomic burdens.

Early life, including the prenatal period and early childhood, is a critical window for neurobehavioral development, during which preventive interventions can be most effective in reducing the risk and severity of these disorders (15, 16). Prenatal nutrition, particularly micronutrients, is essential in fetal neural development with long-term health implications (17). However, micronutrient deficiencies remain widespread among pregnant women globally, including in China (18–20). While some evidence suggests that prenatal iron supplementation may enhance neurobehavioral outcomes (21, 22), other studies have failed to confirm this effect (23). Similarly, randomized controlled trials (RCTs) on prenatal vitamin D supplementation have reported inconsistent results, with some showing improved motor development (24), and others finding no significant benefits (25, 26). For prenatal iodine supplementation, although certain studies suggest cognitive benefits (27, 28), a systematic review of RCTs found no impact on neurobehavioral outcomes (29). These discrepancies may be attributed to heterogeneity in study design, including confounding biases and non-standardized neurobehavioral assessments (25, 26). Therefore, further research using large sample sizes, rigorous control of confounders, and standardized assessment methods is necessary to clarify these associations.

Gut microbiota establishment and neural development share the same critical time window in early life (30). Increasing evidence suggests that gut microbiota influences neurobehavioral development via the gut-brain axis, involving mechanisms such as neurotransmitter regulation, immune modulation, production of neuroactive metabolites (e.g., short-chain fatty acids), and stress response regulation (31–34). Probiotics, which are live, nonpathogenic microorganisms that promote gastrointestinal microbial balance, have been proposed as a potential intervention to enhance neurobehavioral development by modulating gut microbiota (35, 36). Some reviews have reported therapeutic effects of childhood probiotic intake on ASD and ADHD (37–39), while an RCT has investigated its potential in preventing ADHD (40). However, some studies have failed to demonstrate significant benefits of childhood probiotic intake for ASD (41). Moreover, existing research has primarily focused on overt neurobehavioral disorders such as ASD and ADHD, whereas evidence remains limited regarding its effects during the early or subclinical stages of neurobehavioral development.

**Figure 1:**
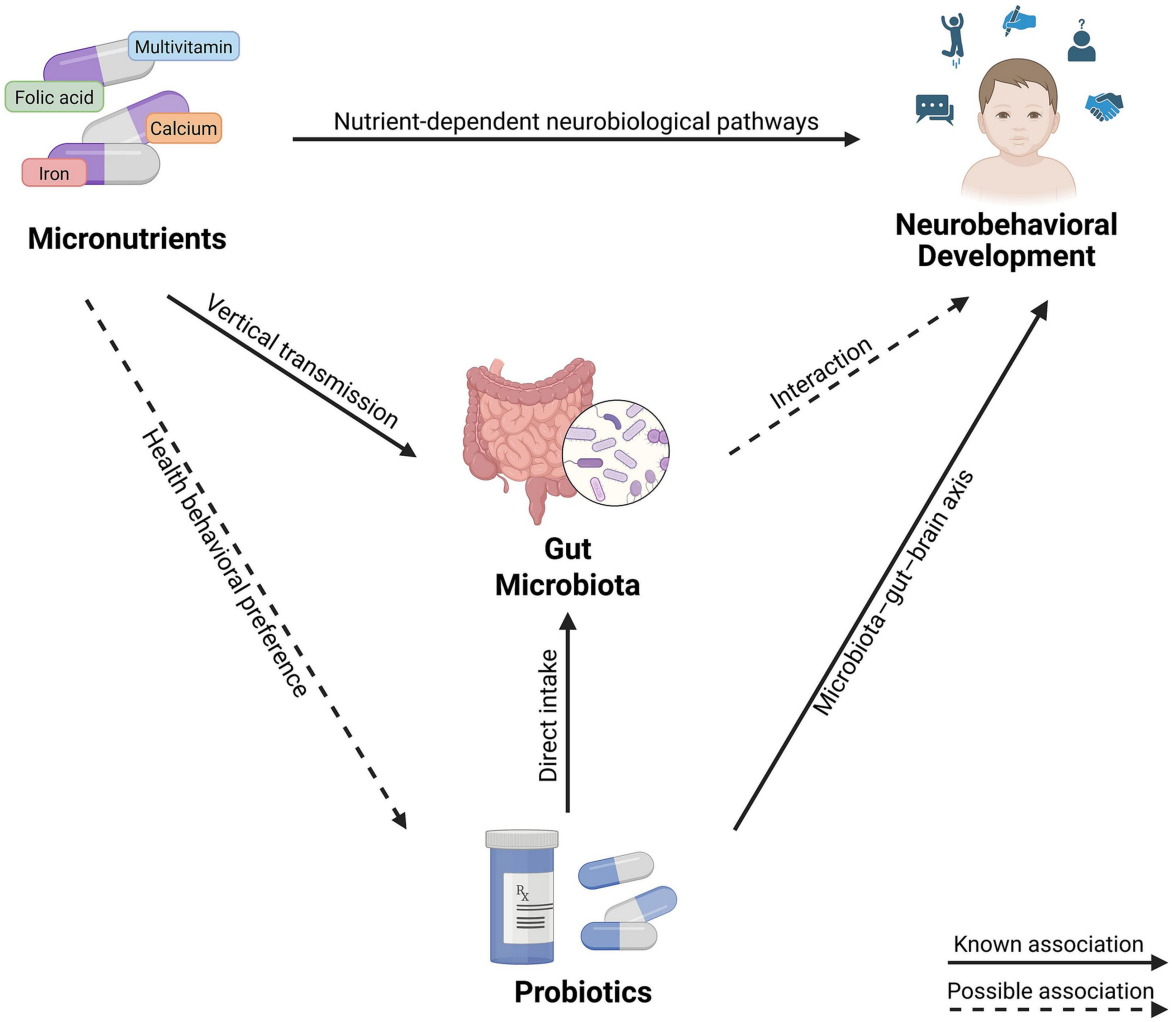
Conceptual framework of the hypothesized associations among prenatal micronutrient supplementation, early childhood probiotic intake, and neurobehavioral development. Created with BioRender.com.

Research shows that prenatal micronutrient supplementation can benefit offspring gut microbiota, while childhood probiotic intake similarly improve gut microbiota composition in children, suggesting a potential interaction between the two in shaping neurobehavioral development via the gut–brain axis (42). Additionally, maternal self-administration of over-the-counter medications during pregnancy may influence the provision of probiotics and other nutritional supplements to their children (43). This indicates that prenatal micronutrient supplementation may also influence childhood probiotic intake, thereby potentially affecting neurobehavioral development. Figure 1 illustrates a conceptual framework of the hypothesized associations among prenatal micronutrient supplementation, early childhood probiotic intake, and neurobehavioral development.

However, existing studies have primarily focused on dietary supplements during a single developmental window—either the prenatal or early childhood. Although some studies have examined the combined effects of prenatal micronutrient supplementation and probiotic intake on maternal and infant outcomes (44–46), the relatively stable maternal microbiota and its indirect influence on the fetus suggest that pregnancy may not be the optimal time for probiotic intake (47). Similarly, while other studies have explored the effects of childhood probiotic intake and micronutrient supplementation (48, 49), initiating micronutrient supplementation during childhood may have limited effects, as neurodevelopment begins in utero and largely depends on maternal nutrient stores (22, 50). Nonetheless, evidence remains limited regarding the effect of childhood probiotic intake on the association between prenatal micronutrient supplementation and neurobehavioral development in children.

Therefore, our study aimed to evaluate the association between prenatal micronutrient supplementation and neurobehavioral development in preschool children, and to explore and quantify the effect of early childhood probiotic intake on this association.

## 2 Methods

### 2.1 Participants

**Figure 2:**
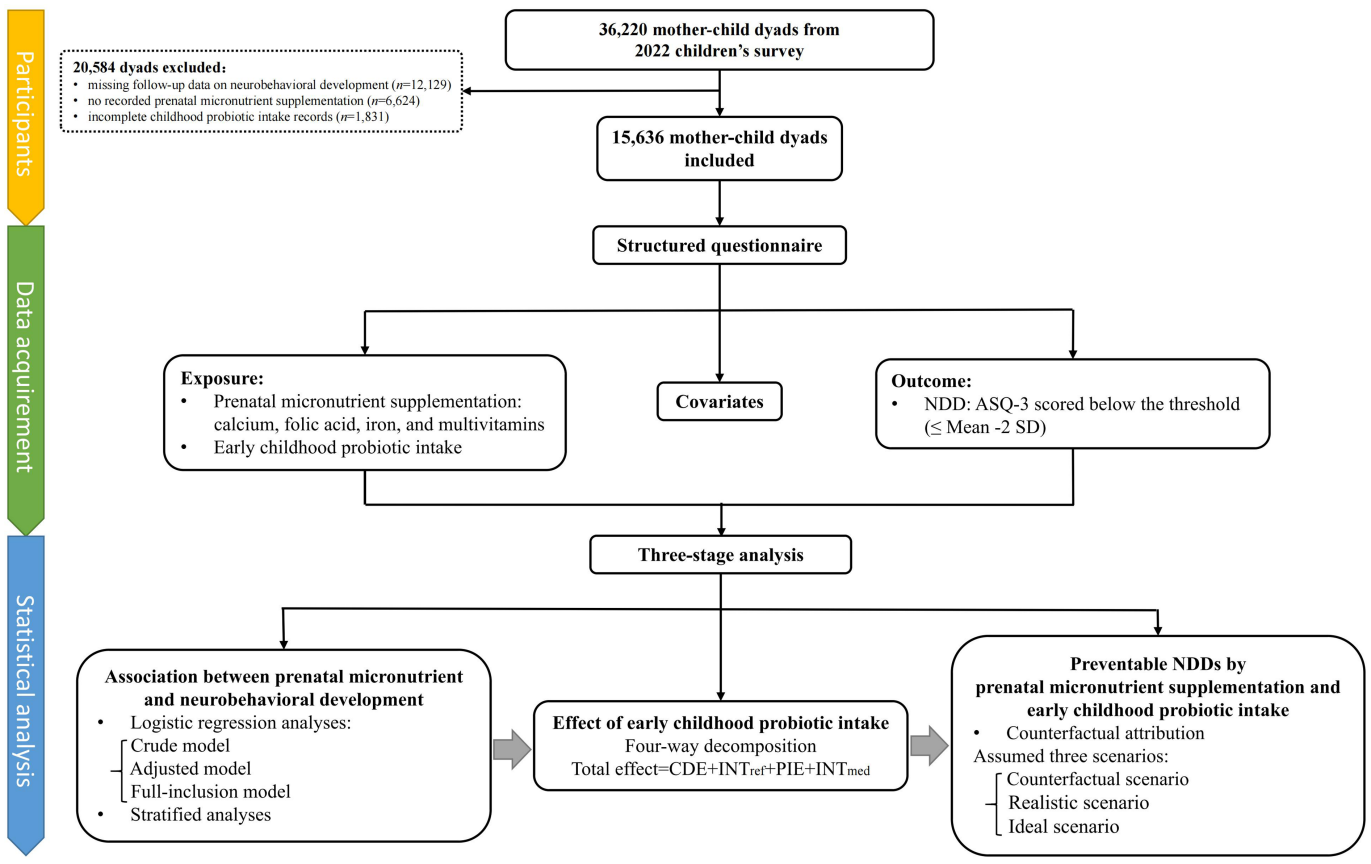
Study profile. ASQ-3, Age and Developmental Progress Questionnaire; CDE, Controlled Direct Effect; INT_med_, mediated interaction; INT_ref_, reference interaction; NDD, neurobehavioral developmental disorder; PIE, Pure Indirect Effect.

The study recruited participants from the 2022 survey of children aged 3–7 years, conducted in 235 kindergartens in Longhua District, Shenzhen, China, with follow-up assessment of neurobehavioral development conducted in 2023. A total of 36,220 mother–child dyads were initially enrolled, and 15,636 participants were included after excluding cases with missing follow-up data on neurobehavioral development (*n* = 12,129), no recorded prenatal micronutrient supplementation (*n* = 6,624), and incomplete childhood probiotic intake records (*n* = 1,831), as shown in Figure 2. Ethical approval was obtained from the Ethics Committee of the School of Public Health, Sun Yat-sen University, and informed consent was provided by the mothers of all participating children.

### 2.2 Data acquirement

Data was collected through a self-administered online structured questionnaire, which was completed by children’s mothers under the supervision of childcare practitioners and kindergarten teachers. The questionnaire was developed by a multidisciplinary panel of epidemiologists, obstetricians, and pediatricians, and its clarity and readability were confirmed through a pilot test. It contained demographic characteristics, maternal condition during pregnancy (e.g., micronutrient supplementation, pregnancy complications, health behaviors), parental lifestyle and health status (e.g., smoking, drinking, diseases), neonatal birth characteristics (e.g., birth weight, preterm birth, delivery mode), lifestyle and health condition at ages 0–3 years (e.g., probiotic intake, feeding pattern, nutritional condition), and children’s health status at ages 3–7 years (e.g., neurobehavioral development, diseases, family function). Further details and coding are provided in Supplementary Table 1.

#### 2.2.1 Prenatal micronutrient supplementation and early childhood probiotic intake

Prenatal micronutrient supplementation (calcium, folic acid, iron, and multivitamins) was assessed through maternal self-reported responses to four separate questions: “Did you take calcium/folic acid/iron/multivitamins during your pregnancy?” Participants answering ‘YES’ were assigned to the corresponding supplementation group, while all others served as the reference group.

Early childhood probiotic intake was defined based on a ‘YES’ response to the question: “Did your child take probiotics between the ages of 0–3 years?” Here, probiotics were specified as a single product form (capsules or sachets) not combined with other foods or supplements.

#### 2.2.2 Outcome

The neurobehavioral development in preschool children (3–7 years old) was assessed by the Age and Developmental Progress Questionnaire-Third Edition (ASQ-3), a well-validated and widely used tool in multiple countries, including China, demonstrating good internal consistency (Cronbach’s *α* = 0.8) (51–53). Designed for children aged 1 to 66 months, the ASQ-3 assesses five key domains: communication, gross motor, fine motor, problem-solving, and personal-social status. Assessment results are classified into three groups: (1) scores above the threshold (>mean minus 1 standard deviation [SD]), indicating age-appropriate development; (2) scores close to the threshold (mean minus 2 SD to mean minus 1 SD), requiring further monitoring; and (3) scores below the threshold (≤mean minus 2 SD), indicating developmental disorder. In our study, the presence of neurobehavioral developmental disorder (NDD) was identified when at least one domain scored below the threshold, while others with all domains above or close to the threshold were classified as neurobehavioral developmental normality (NDN).

#### 2.2.3 Covariates

Based on previous studies (21, 27, 54–56) and the univariate and multivariate analyses results (See Supplementary Table 2), covariates included child’s demographic characteristics (age, sex, birth season, residence type), maternal demographic characteristics (education, household income, age of conception, pre-pregnancy BMI), pregnancy and perinatal characteristics (intrauterine growth restriction [IUGR], parity, preterm birth [PTB], and birth weight [BW]), and childhood family environment (parental depression, family functioning, feeding pattern). The average missing data rate for these covariates was 3.9% (range: 0–12.9%), with missing data addressed using multiple imputations through Predictive Mean Matching (PMM) (57).

### 2.3 Statistical analysis

We compared variables between the NDD and NDN groups using one-way ANOVA and t-tests for continuous variables and chi-square tests for categorical variables. The main analysis was conducted in three stages.

#### 2.3.1 Association between prenatal micronutrient supplementation and neurobehavioral development in preschool children

We examined the association between maternal micronutrient supplementation during pregnancy and neurobehavioral development in preschool children using univariate and multivariate logistic regression analyses under three different models: the crude model, without adjustment for confounders; the adjusted model, adjusted for selected confounders; and the full-inclusion model, included all micronutrients in the model while adjusting for all confounders to address the confounding effects of co-supplementation. To further explore the association between prenatal micronutrient supplementation and neurobehavioral development under different probiotic intake scenarios, we conducted a stratified analysis based on probiotic intake in early childhood.

#### 2.3.2 Effect of early childhood probiotic intake on association between prenatal micronutrient supplementation and neurobehavioral development in preschool children

Under the three models, we analyzed the effect of probiotic intake on the association between prenatal micronutrient supplementation and neurobehavioral development in preschool children through four-way decomposition, as proposed by J. VanderWeele (58). This method allows for simultaneous analysis of interaction and mediation effects, which is widely used in epidemiologic studies (59–61). The core is to decompose the total effect into four components: (1) controlled direct effect (CDE): the effect of prenatal micronutrient supplementation on neurobehavioral development independent of childhood probiotic intake, (2) reference interaction (INT_ref_): the effect of prenatal micronutrient supplementation on neurobehavioral development only through its interaction with childhood probiotic intake, (3) pure indirect effect (PIE): the effect of prenatal micronutrient supplementation on neurobehavioral development both through an interaction with and mediation by childhood probiotic intake, and (4) mediated interaction (INT_med_): the effect of prenatal micronutrient supplementation on neurobehavioral development only through the mediation by childhood probiotic intake. Estimates of the four components were obtained through regression analyses that included the exposure, mediator, and their interaction terms, with results presented as excess odds ratio (EOR) and proportion attributable (PA).

#### 2.3.3 Preventable NDD by prenatal micronutrient supplementation and early childhood probiotic intake

After identifying the effect of probiotic intake, we quantified the number of preventable NDD attributable to prenatal micronutrient supplementation and early childhood probiotic intake using counterfactual attribution (62), which can be used to quantify interaction or mediation effects (63). We assumed three scenarios: (1) Counterfactual scenario (V1): no probiotic intake, (2) Realistic scenario (V2): probiotic intake in the realistic proportion of the survey population, and (3) Ideal scenario (V3): probiotic intake in all populations. Under each scenario, we estimated the effect of prenatal micronutrient supplementation and early childhood probiotic intake on NDD across three models to calculate the number of preventable NDD cases. The difference in preventable cases between the realistic and counterfactual scenarios (V2–V1) represented the additional preventable NDD due to current childhood probiotic intake proportion, while the difference between the ideal and counterfactual scenarios (V3–V1) represents the potential preventable NDD if probiotics were consumed by all populations.

Statistical analyses above were performed via R version 4.2.3. Two sided *p*-values <0.05 were considered significant.

## 3 Results

### 3.1 Participants’ characteristics

Table 1 presents the background characteristics of the study participants, including prenatal micronutrient supplementation and childhood probiotic intake, with a comparison between NDD and NDN groups. Folic acid (88.2%) was the most widely consumed, followed by calcium (75.8%), while iron (46.0%) and multivitamin supplementation (44.4%) were relatively less common in pregnant women. A total of 80.2% of children reported taking probiotics in early childhood.

**Table 1:** Background characteristics of study participants in the 2022 children’s survey.

Characteristic	Overall^a^ (*n* = 15,636)	Outcome		*p*-value^b^
		NDD^a^ (*n* = 13,804)	NDN^a^ (*n* = 1,832)	
Child’s age	4.6 ± 0.6	4.6 ± 0.6	4.6 ± 0.5	<0.001
Child’s sex				<0.001
Male	8,346 (53.4%)	7,219 (52.3%)	1,127 (61.5%)	
Female	7,290 (46.6%)	6,585 (47.7%)	705 (38.5%)	
Birth season				<0.001
Spring	4,225 (27.0%)	3,746 (27.1%)	479 (26.1%)	
Summer	4,482 (28.7%)	3,845 (27.9%)	637 (34.8%)	
Autumn	2,945 (18.8%)	2,618 (19.0%)	327 (17.8%)	
Winter	3,984 (25.5%)	3,595 (26.0%)	389 (21.2%)	
Residence type				<0.001
Shenzhen residents	9,415 (60.2%)	8,507 (61.6%)	908 (49.6%)	
Non-Shenzhen residents	6,221 (39.8%)	5,297 (38.4%)	924 (50.4%)	
Maternal education				<0.001
Less than high school	1,614 (10.3%)	1,262 (9.14%)	352 (19.2%)	
High school and higher	14,022 (89.7%)	12,542 (90.9%)	1,480 (80.8%)	
Household income				<0.001
<RMB 20,000	7,314 (46.8%)	6,262 (45.4%)	1,052 (57.4%)	
≥RMB 20,000	8,322 (53.2%)	7,542 (54.6%)	780 (42.6%)	
Maternal conception age	34.0 ± 5.5	34.0 ± 5.5	33.7 ± 5.7	0.028
Pre-pregnancy BMI				<0.001
BMI < 18.5	2,890 (18.5%)	2,550 (18.5%)	340 (18.6%)	
18.5 ≤ BMI < 24	10,571 (67.6%)	9,392 (68.0%)	1,179 (64.4%)	
BMI ≥ 24	2,175 (13.9%)	1,862 (13.5%)	313 (17.1%)	
Intrauterine growth retardation				<0.001
No	15,497 (99.1%)	13,697 (99.2%)	1,800 (98.3%)	
Yes	139 (0.9%)	107 (0.8%)	32 (1.7%)	
Parity				0.21
Nulliparous	8,810 (56.3%)	7,752 (56.2%)	1,058 (57.8%)	
Multiparous	6,826 (43.7%)	6,052 (43.8%)	774 (42.2%)	
Preterm birth				<0.001
No	14,517 (92.8%)	12,851 (93.1%)	1,666 (90.9%)	
Yes	1,119 (7.2%)	953 (7.0%)	166 (9.1%)	
Child’s birth weight	3.1 ± 0.6	3.1 ± 0.6	3.0 ± 0.7	<0.001
Parental depression				<0.001
No	13,652 (87.3%)	12,140 (87.9%)	1,512 (82.5%)	
Yes	1,984 (12.7%)	1,664 (12.1%)	320 (17.5%)	
Family function				<0.001
Normal	9,697 (62.0%)	8,772 (63.5%)	925 (50.5%)	
Dysfunction	5,939 (38.0%)	5,032 (36.5%)	907 (49.5%)	
Feeding pattern				<0.001
Breastfeeding	8,803 (56.3%)	7,815 (56.6%)	988 (53.9%)	
Formula feeding	1,665 (10.6%)	1,415 (10.3%)	250 (13.6%)	
Mixed feeding	5,168 (33.1%)	4,574 (33.1%)	594 (32.4%)	
Prenatal calcium supplementation				0.003
No	3,781 (24.2%)	3,286 (23.8%)	495 (27.0%)	
Yes	11,855 (75.8%)	10,518 (76.2%)	1,337 (73.0%)	
Prenatal folic acid supplementation				0.013
No	1,846 (11.8%)	1,597 (11.6%)	249 (13.6%)	
Yes	13,790 (88.2%)	12,207 (88.4%)	1,583 (86.4%)	
Prenatal iron supplementation				0.11
No	8,451 (54.0%)	7,428 (53.8%)	1,023 (55.8%)	
Yes	7,185 (46.0%)	6,376 (46.2%)	809 (44.2%)	
Prenatal multivitamin supplementation				<0.001
No	8,690 (55.6%)	7,549 (54.7%)	1,141 (62.3%)	
Yes	6,946 (44.4%)	6,255 (45.3%)	691 (37.7%)	
Childhood probiotic intake				0.010
No	3,098 (19.8%)	2,777 (20.1%)	321 (17.5%)	
Yes	12,538 (80.2%)	11,027 (79.9%)	1,511 (82.5%)	

a Data are presented as Mean ± SD or *N* (%).

b *p*-value was based on one-way analysis of means and Pearson’s Chi-squared test where appropriate.

The overall average ASQ-3 score of the 15,636 children was 277 ± 26.4, with domain-specific scores ranging from 51.7 ± 10.2 in the fine motor domain to 57.6 ± 5.4 in the communication domain. A total of 11.7% of the children were identified as NDD, with the highest prevalence in the gross motor domain (8.88%) and the lowest in the problem-solving domain (0.70%), as shown in Table 2.

**Table 2:** Neurobehavioral development across five domains in the 2022 children’s survey.

Domains	Description (*n* = 15,636)	
	Score, Mean ± SD	Prevalence, *n* (%)
Communication	57.6 ± 5.4	
Normal		15,457 (98.9%)
Disorder		179 (1.1%)
Gross motor	54.2 ± 8.6	
Normal		14,248 (91.1%)
Disorder		1,388 (8.9%)
Fine motor	51.7 ± 10.2	
Normal		15,214 (97.3%)
Disorder		422 (2.7%)
Problem solving	57.2 ± 5.7	
Normal		15,526 (99.3%)
Disorder		110 (0.7%)
Personal-social	56.8 ± 5.6	
Normal		15,281 (97.7%)
Disorder		355 (2.3%)
Total	277.5 ± 26.4	
Normal		13,804 (88.3%)
Disorder		1,832 (11.7%)

### 3.2 Association between prenatal micronutrient supplementation and neurobehavioral development in preschool children

**Figure 3:**
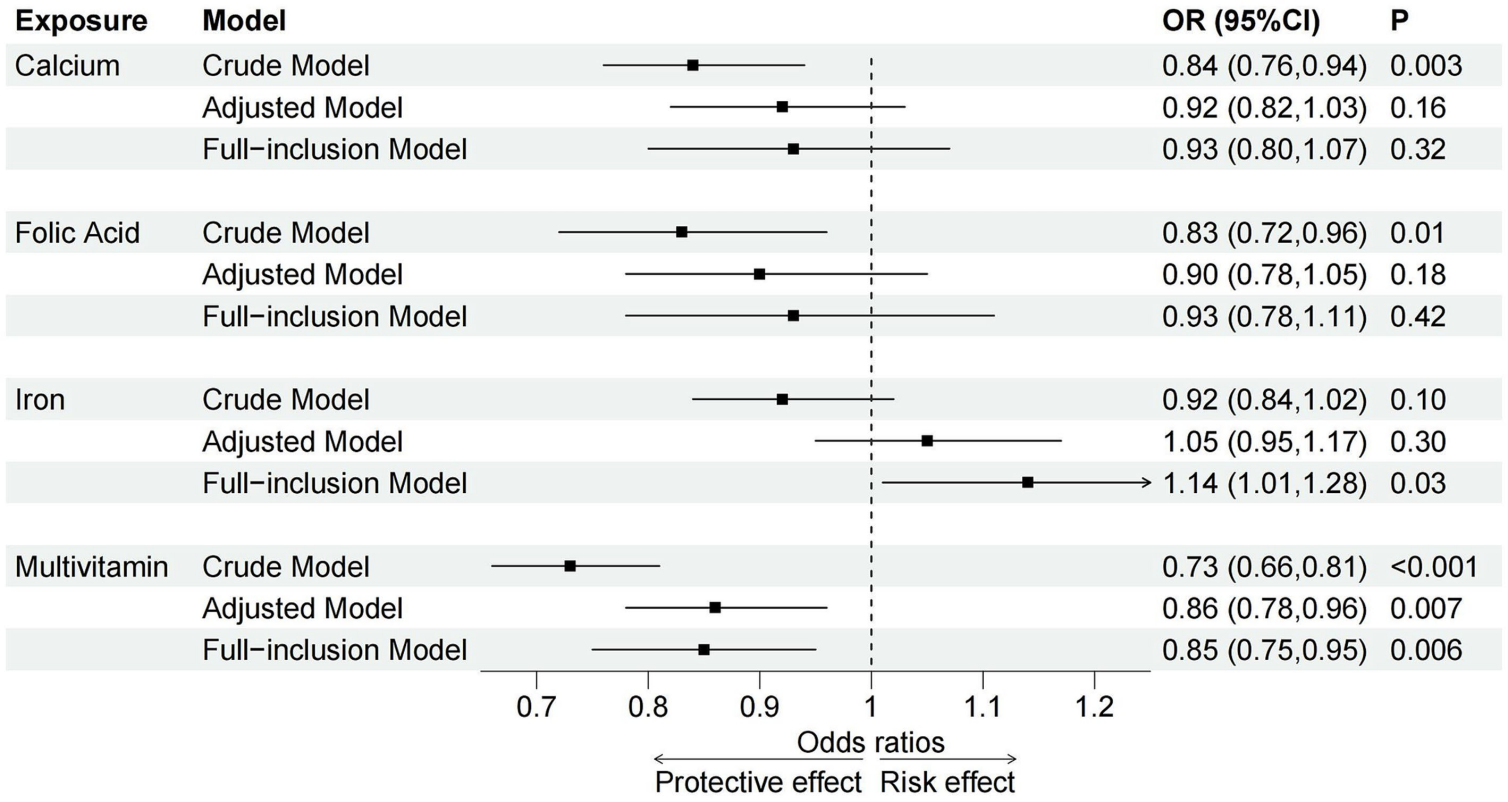
Associations between prenatal micronutrient supplementation and NDD in crude, adjusted and full-inclusion models. OR, odds ratio; CI, confidence interval; P, *p*-value.

In the crude model, prenatal calcium (OR = 0.84, 95% CI = 0.76–0.94), folic acid (OR = 0.83, 95% CI = 0.72–0.96) and multivitamin (OR = 0.73, 95% CI = 0.66–0.81) supplementation was associated with a decreased risk of NDD, whereas no significant association was observed for iron. In the adjusted and full-inclusion models, only prenatal multivitamin supplementation remained associated with a reduced risk of NDD (Adjusted Model: OR = 0.86, 95% CI = 0.78–0.96; Full-inclusion Model: OR = 0.85, 95% CI = 0.75–0.95) (Supplementary Table 3; Figure 3). In the full-inclusion model, prenatal iron supplementation was even found to be significantly associated with an increased risk of NDD (OR = 1.14, 95% CI = 1.01–1.28). Across these 5 domains, after controlling for confounders, prenatal calcium and folic acid supplementation were significantly associated with NDD in the communication and gross motor domains, folic acid and multivitamin supplementation were associated with NDD in the personal-social domain, whereas no significant associations were found in the fine motor and problem-solving domains (Supplementary Table 4).

**Figure 4:**
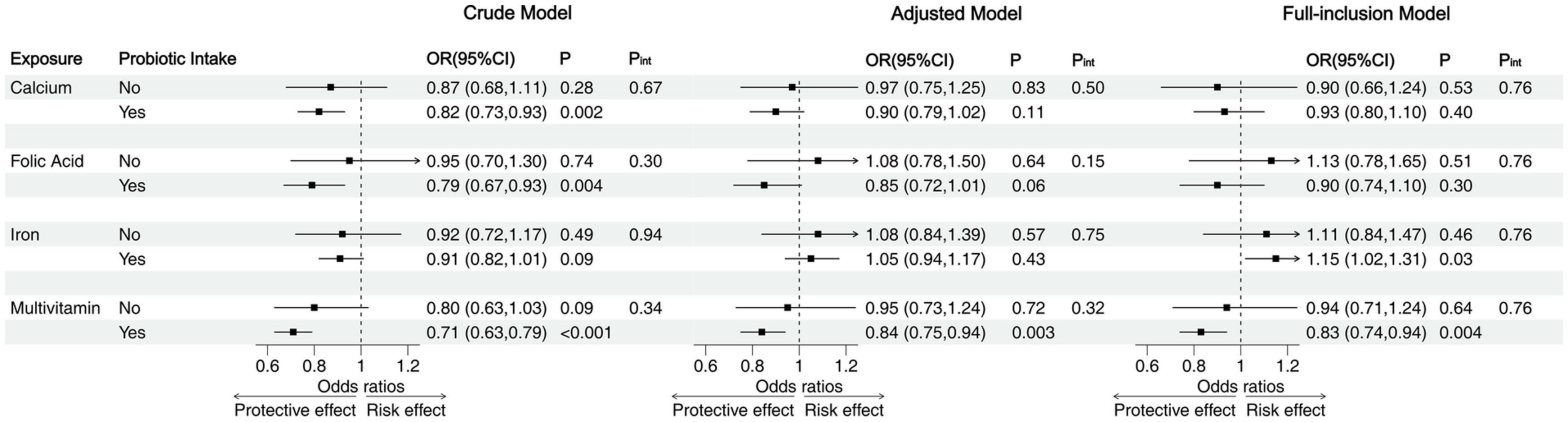
Stratified analysis of the associations between prenatal micronutrient supplementation and NDD by childhood probiotic intake. OR, odds ratio; CI, confidence interval; P, *p*-value; P_int_, *p*-value for the interaction between prenatal micronutrient supplementation and childhood probiotic intake.

When the analysis was stratified by probiotic intake, we found that prenatal micronutrient supplementation was not significantly associated with NDD without childhood probiotic intake. However, with childhood probiotic intake, the association between prenatal micronutrient supplementation and NDD followed the same pattern as in the whole sample, with multivitamin supplementation associated with a decreased risk of NDD across all three models (Crude model: OR = 0.71, 95% CI = 0.63–0.79; Adjusted Model: OR = 0.84, 95% CI = 0.75–0.94; Full-inclusion Model: OR = 0.83, 95% CI = 0.74–0.94). Although no significant differences were observed between the probiotic and non-probiotic intake subgroups, most ORs were lower in the probiotic intake subgroup, with many associations showing statistical significance within this subgroup only (Figure 4). According to the stratified analysis in the five domains, compared to the non-probiotic intake subgroup, prenatal folic acid and multivitamin supplementation were significantly associated with reduced risks in the gross motor and personal-social domains in the probiotic intake group. In the communication, fine motor and problem-solving domains, though lower ORs were observed, none showed statistical significance in the probiotic intake group (Supplementary Table 5).

### 3.3 Effect of early childhood probiotic intake on association between prenatal micronutrient supplementation and neurobehavioral development in preschool children

**Figure 5:**
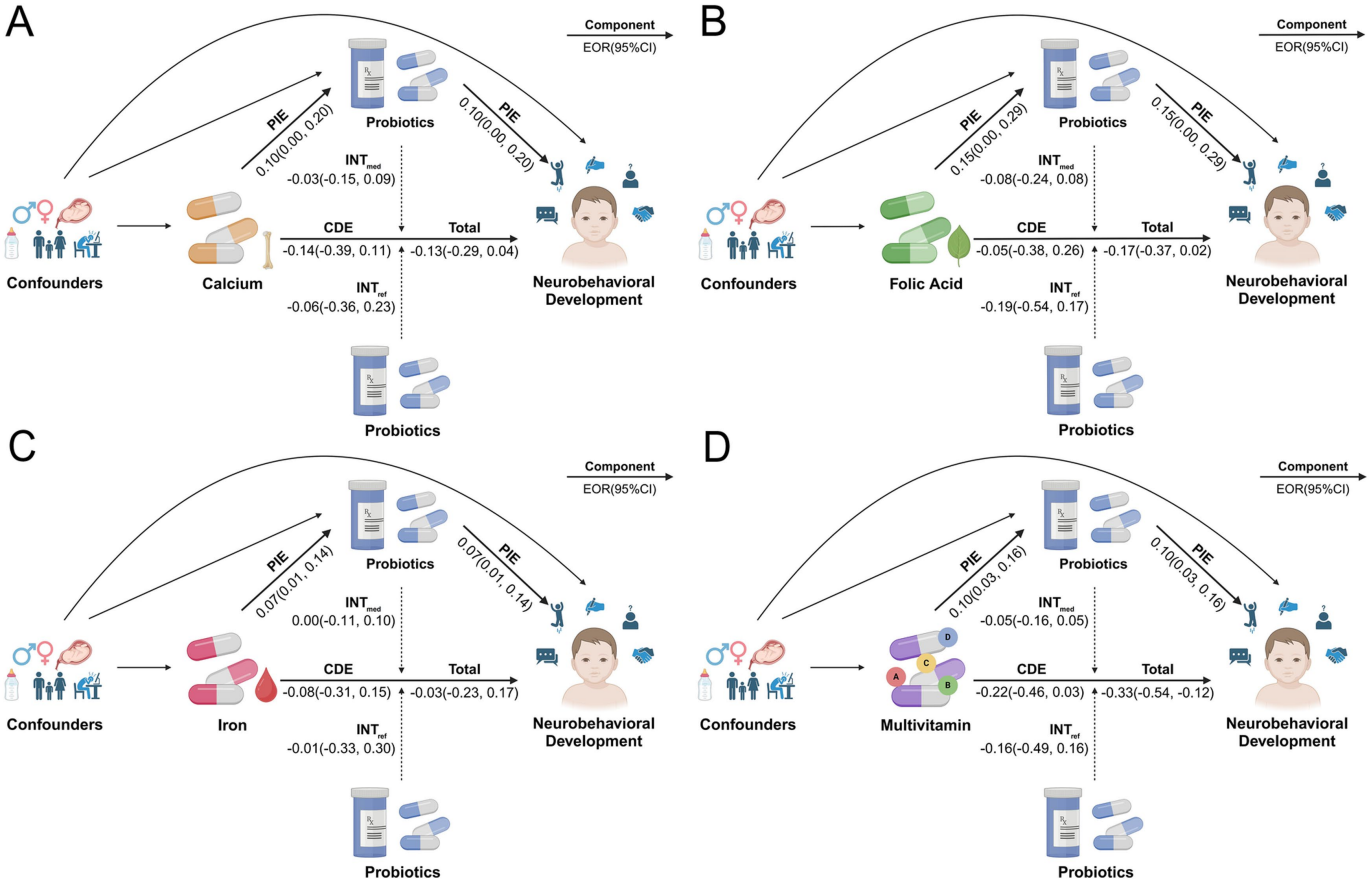
Four-way decomposition diagram of the effect of childhood probiotic intake on the association between prenatal micronutrient supplementation and NDD in the crude model. **(A)** Calcium supplementation; **(B)** Folic acid supplementation; **(C)** Iron supplementation; **(D)** Multivitamin supplementation. CDE, controlled direct effect; CI, confidence interval; EOR, excess odds ratio; INT_med_, mediated interaction; INT_ref_, reference interaction; OR, odds ratio; PIE, pure indirect effect. Created with BioRender.com.

In the crude model, the results showed that childhood probiotic intake was significantly associated with an enhanced protective effect of prenatal multivitamin supplementation on NDD (Total EOR = −0.33, 95% CI = −0.54 to−0.12 vs. CDE EOR = −0.22, 95% CI = −0.46 to 0.03). Most of this protective effect from childhood probiotic intake was driven by the INT_ref_ (EOR = −0.16, 95% CI = −0.49 to 0.16), accounting for 48% of the total effect. The significant mediating effect of probiotic intake was also observed (EOR = 0.10, 95% CI = 0.03–0.16). The INT_med_ followed (EOR = −0.05, 95% CI = −0.16 to 0.05), suggesting the presence of both interaction and mediation of childhood probiotic intake. No significant enhanced protective effects by childhood probiotic intake were observed in relation to prenatal supplementation of calcium, folic acid or iron (Figure 5; Table 3). Across the five domains, childhood probiotic intake was significantly associated with an enhanced protective effect of prenatal multivitamin supplementation on disorders in gross motor development (Total EOR = −0.31, 95% CI = −0.56 to −0.07) and personal-social development (Total EOR = −0.50, 95% CI = −0.97 to −0.03). The effect of prenatal folic acid supplementation on disorders of personal-social development was also significantly increased by childhood probiotic intake (Total EOR = −0.51, 95% CI = −0.88 to −0.14) (Supplementary Table 6).

**Table 3:** The effect of childhood probiotic intake on the association between prenatal micronutrient supplementation and NDD using the four-way decomposition.

Component	Crude model		Adjusted model		Full-inclusion model	
	EOR (95%CI)	PA (95%CI), %	EOR (95%CI)	PA (95%CI), %	EOR (95%CI)	PA (95%CI), %
Calcium supplementation						
*CDE*	−0.14 (−0.39, 0.11)	107 (304, −89)	−0.01 (−0.27, 0.23)	7 (156, −135)	0.00 (−0.27, 0.27)	−3 (163, −165)
*INTref*	−0.06 (−0.36, 0.23)	50 (284, −179)	−0.19 (−0.75, 0.40)	111 (437, −232)	−0.18 (−0.71, 0.37)	111 (435, −226)
*INT_med_*	−0.03 (−0.15, 0.09)	20 (115, −73)	−0.04 (−0.16, 0.08)	23 (91, −48)	−0.02 (−0.07, 0.04)	10 (41, −22)
*PIE*	0.10 (0.00, 0.20)	−78 (−1, −157)	0.07 (−0.03, 0.16)	−40 (19, −96)	0.03 (−0.02, 0.07)	−18 (11, −45)
Total	−0.13 (−0.29, 0.04)	100	−0.17 (−0.58, 0.24)	100	−0.16 (−0.53, 0.21)	100
Folic acid supplementation						
*CDE*	−0.05 (−0.38, 0.26)	30 (218, −150)	0.09 (−0.23, 0.40)	−23 (58, −99)	0.14 (−0.21, 0.47)	−42 (63, −144)
*INT_ref_*	−0.19 (−0.54, 0.17)	109 (309, −100)	−0.52 (−1.22, 0.22)	129 (302, −54)	−0.47 (−1.17, 0.24)	144 (359, −73)
*INT_med_*	−0.08 (−0.24, 0.08)	48 (137, −45)	−0.10 (−0.25, 0.05)	26 (61, −12)	−0.02 (−0.08, 0.03)	8 (24, −9)
*PIE*	0.15 (0.00, 0.29)	−86 (−2, −169)	0.13 (−0.02, 0.26)	−32 (4, −64)	0.03 (−0.03, 0.09)	−9 (9, −28)
Total	−0.17 (−0.37, 0.02)	100	−0.40 (−0.89, 0.10)	100	−0.33 (−0.79, 0.13)	100
Iron supplementation						
*CDE*	−0.08 (−0.31, 0.15)	298 (1,110, −520)	0.08 (−0.16, 0.33)	446 (−841, 1,754)	0.17 (−0.08, 0.42)	155 (−79, 391)
*INT_ref_*	−0.01 (−0.33, 0.30)	43 (1,176, −1,068)	−0.09 (−0.66, 0.48)	−478 (−3,486, 2,536)	−0.08 (−0.59, 0.44)	−72 (−551, 407)
*INT_med_*	0.00 (−0.11, 0.10)	14 (380, −347)	−0.02 (−0.12, 0.09)	−88 (−650, 473)	−0.01 (−0.07, 0.05)	−9 (−66, 48)
*PIE*	0.07 (0.01, 0.14)	−255 (−22, −494)	0.04 (−0.03, 0.11)	220 (−134, 560)	0.03 (−0.01, 0.07)	26 (−12, 63)
Total	−0.03 (−0.23, 0.17)	100	0.02 (−0.41, 0.45)	100	0.11 (−0.24, 0.45)	100
Multivitamin supplementation						
*CDE*	−0.22 (−0.46, 0.03)	65 (137, −9)	−0.03 (−0.28, 0.22)	10 (85, −66)	−0.04 (−0.30, 0.23)	11 (95, −74)
*INT_ref_*	−0.16 (−0.49, 0.16)	48 (148, −49)	−0.30 (−0.91, 0.30)	92 (273, −92)	−0.28 (−0.81, 0.26)	89 (261, −83)
*INT_med_*	−0.05 (−0.16, 0.05)	15 (48, −16)	−0.06 (−0.18, 0.06)	18 (54, −18)	−0.04 (−0.13, 0.04)	14 (42, −14)
*PIE*	0.10 (0.03, 0.16)	−29 (−10, −48)	0.07 (0.00, 0.13)	−20 (1, −40)	0.05 (−0.01, 0.10)	−15 (2, −32)
Total	−0.33 (−0.54, −0.12)	100	−0.33 (−0.80, 0.15)	100	−0.31 (−0.68, 0.07)	100

### 3.4 Preventable NDD by prenatal micronutrient supplementation and childhood probiotic intake

**Figure 6:**
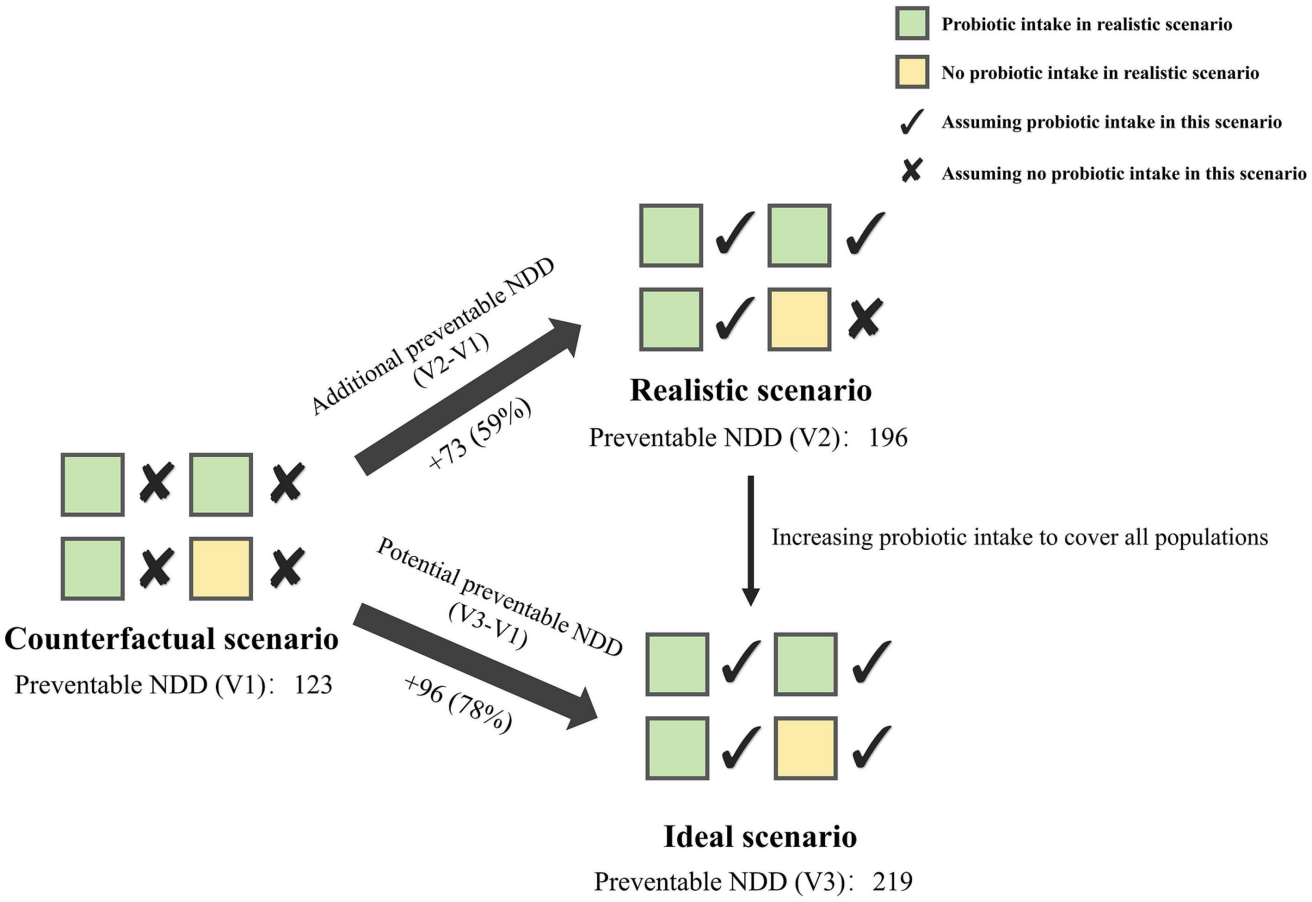
Preventable NDD by prenatal multivitamin supplementation and childhood probiotic intake in the crude model. Counterfactual scenario: without probiotic intake; Realistic scenario: with probiotic intake in the realistic proportion of the survey population; Ideal scenario: with probiotic intake in all populations. NDD, neurobehavioral developmental disorder.

In the crude model where a significant effect of childhood probiotic intake was observed on the association between prenatal multivitamin supplementation and NDD, we assumed three scenarios: In the scenario assuming no probiotic intake in early childhood (counterfactual scenario), among the 15,636 participants, prenatal multivitamin supplementation alone could prevent 123 children from developing NDD. Under current childhood probiotic intake proportion (realistic scenario), 73 additional NDD cases could be prevented compared to no probiotic intake, representing a 59% increase in preventive effect. If childhood probiotic intake were increased to a scenario where all children consume probiotics (ideal scenario), it could potentially prevent 96 more children from developing NDD compared to no probiotic intake, representing a 78% increase (Figure 6).

The highest number of preventable NDD was in the gross motor domain (47 cases in the realistic scenario and 64 in the ideal scenario), followed by the personal-social domain (19 in the realistic scenario and 24 in the ideal scenario). The highest percentage increase in NDD prevention was observed in the fine motor domain, with a 92% increase in the realistic scenario and a 117% increase in the ideal scenario, followed by the personal-social domain (80% in the realistic and 100% in the ideal scenario) (Table 4). The number of preventable NDD across the five domains under different models is detailed in Supplementary Table 7. Most prenatal micronutrient supplementation with childhood probiotic intake showed varied increases in NDD prevention in both realistic and ideal scenarios.

**Table 4:** Number of preventable NDD by prenatal multivitamin supplementation and childhood probiotic intake in counterfactual, realistic, and ideal scenario in the crude model.

Domains	Counterfactual scenario	Realistic scenario			Ideal scenario		
	Preventable NDD (V1)	Preventable NDD (V2)	Additional preventable NDD (V2–V1)	Additional preventable percentage, %	Preventable NDD (V3)	Potential preventable NDD (V3–V1)	Potential preventable percentage, %
Communication	25	26	2	7	27	3	11
Gross motor	109	156	47	43	173	64	59
Fine motor	17	32	15	92	36	20	117
Problem solving	13	14	0	3	13	0	−2
Personal-Social	24	44	19	80	49	24	100
Total	123	196	73	59	219	96	78

## 4 Discussion

Our study revealed that 11.7% of preschool children were identified with NDD in the 2022 children’s survey. We found that prenatal multivitamin supplementation was significantly associated with a reduced risk of NDD across the crude, adjusted and full-inclusion models. When exploring the effect of probiotic intake in early childhood, our results indicated that childhood probiotic intake was associated with an enhanced protective effect of prenatal multivitamin supplementation against NDD in the crude model (Total EOR = −0.33, 95% CI = −0.54 to 0.12), with 48% of the effect attributable to interactions. Quantifying this enhanced protective effect, our study demonstrated that childhood probiotic intake contributed to the prevention of 73 (a 59% increase) additional NDD cases, with the potential to prevent 96 additional NDD cases (a 78% increase) if childhood probiotic intake were consumed by all populations.

Previous research have found that prenatal single vitamin supplementation, such as vitamins D and B12, are associated with a lower risk of NDD in children (25, 64–66), while multivitamins also significantly promote neurobehavioral development in children (67, 68), which is consistent with the results of this study. Vitamins are essential for fetal brain development, serving as cofactors in neurotransmitter synthesis and enzymatic metabolism processes (69). For instance, vitamin B12 is crucial for fatty acid metabolism necessary for myelin sheath production, while vitamin B6 functions as a coenzyme in the synthesis of various amino acid neurotransmitters, both of which can influence neurobehavioral development (70, 71). Retinoids, derived from vitamin A, contribute to neuronal differentiation and influence functions like memory and sleep (72). Some evidence suggests that multivitamin supplementation exert broader effects on neurobehavioral development because they allow multiple biological pathways for effects (71). However, other studies indicated that multivitamin supplementation not always more effective than single vitamins for cognitive function (73). Further research is warranted to clarify the comparative benefits of multivitamin supplementation versus single-vitamin supplementation in neurobehavioral development. Despite the known benefits of vitamins, our study found that fewer than half of pregnant women took multivitamins, as reported in other surveys, highlighting a need to improve multivitamin supplementation practices (66).

Folic acid, widely recognized as an important substance in neural tube development, is supplemented at a higher prevalence, possibly because of its inclusion in the WHO’s list of essential medicines for pregnant women (74–76). Similar to our findings, studies have shown that prenatal folic acid supplementation is positively associated with children’s neurobehavioral development (77, 78). However, some reports suggest that excessive folic acid intake may increase the risk of ASD and food allergies, indicating a U-shaped association (79, 80). This underscores the importance of determining precise supplementation levels, especially given that nearly all prenatal foods already contain increased levels of folic acid (66). Iron and calcium, involved in neurotransmitter function, energy metabolism, and myelination, may also influence neurobehavioral development (81, 82). However, our study did not find a significant association between prenatal calcium supplementation and neurobehavioral outcomes, and even identified iron supplementation as a risk factor. Similar conclusions have been drawn in other population studies (83, 84). Variability in study populations, research design, exposure timing and neurobehavioral assessment tools may account for these results (84, 85).

Our findings further suggest that childhood probiotic intake is associated with an enhanced protective effect of prenatal multivitamin supplementation against NDD, primarily through their interaction, as suggested by previous reviews (86, 87). On the one hand, vitamins benefit maternal gut microbiota, with vitamin A and B2 increasing microbial diversity and abundance and vitamins A and D maintaining intestinal barrier integrity (88–90). Beneficial maternal microbes can be transferred to offspring through the birth canal, breast milk, and even vertically in utero (42, 91–93). Probiotic intake in early childhood may further enhance the colonization and function of these inherited beneficial microbiota, improving neurobehavioral outcomes through the gut-brain axis (55, 94). On the other hand, gut microbiota can synthesize specific vitamins such as vitamin K and the B vitamins (95), which can improve cognitive function and reduce the risk of NDD in multiple pathways (96, 97). These findings reinforce the idea that maternal nutrition during pregnancy interacts with offspring gut microbiota to influence neurobehavioral development. Additionally, our study also found that probiotics acted as a reverse mediator in the relationship between micronutrients and NDD, potentially increasing the risk of NDD. One possible explanation is that mothers who self-medicate during pregnancy are more likely to administer probiotics to their children (43), often to address gut microbiota imbalances. Such pre-existing gut microbiota imbalances in children may adversely affect neurobehavioral development through oxidative stress (98).

Quantifying the enhanced protective effect of childhood probiotic intake on NDD, we found the effect was particularly present in gross motor, fine motor and personal-social developmental disorders. The reason may be that the influence of gut microbiota on the brain is mainly concentrated in the limbic system and motor cortex, which are related to emotion and motor coordination, while its effects on the prefrontal cortex and hippocampus, which are associated with problem-solving, are more indirect (99, 100). This result also echoes the protective effects of probiotic intake against Parkinson’s disease, which is associated with fine motor disorders, and ASD, which is associated with emotional–social dysfunction (101, 102). In addition, we also observed that early childhood probiotic intake significantly increased the number of gross motor developmental disorders prevented by prenatal multivitamin supplementation, which happened to be the most prevalent type of NDD in our study. This suggests that early childhood probiotic intake can specifically target the prevention of neurobehavioral developmental domains that most need improvement.

This study makes several significant contributions. First, it innovatively introduces early childhood probiotic intake as a key variable to explore its effect on the association between prenatal micronutrient supplementation and neurobehavioral development in children, addressing the limitations of previous studies focused on single time windows. Second, the study employs advanced statistical techniques, including four-way decomposition and counterfactual mediation analysis, to systematically evaluate the potential effect of probiotic intake from different perspectives. Finally, the study is based on a large-scale children’s survey, enhancing the reliability and generalizability of the findings.

Nevertheless, the study has several limitations. First, as all participants were recruited from Shenzhen, China, our findings may not be generalizable to other populations. Second, data collection relied on retrospective questionnaires, which may introduce recall bias. Additionally, ASQ-3 assessments, based on maternal reports, may be subject to reporting bias compared to clinical diagnoses. Moreover, prenatal micronutrient supplementation and childhood probiotic intake were recorded as binary variables, lacking detailed dosage information and probiotic strain data. Although we adjusted for several factors that may reflect maternal health consciousness on dietary supplement use, the potential for self-selection bias remains. Lastly, although we controlled for confounders, the cross-sectional study design limits causal inference.

Therefore, future studies should focus on determining specific supplementation dosages to establish a dose–response relationship, providing clearer supplementation guidelines. Higher-evidence studies, such as RCTs, are needed to confirm the causal relationship between prenatal micronutrient supplementation, childhood probiotic intake, and NDD. In addition, molecular-level research is essential to elucidate the biological mechanisms underlying these effects and to explore the complex pathways influencing different neurobehavioral domains.

## 5 Conclusion

In summary, our study found that prenatal multivitamin supplementation has a protective effect against NDD in preschool children. Early childhood probiotic intake is associated with an enhancement of this protective effect, primarily driven by interaction with prenatal multivitamin supplementation. Early childhood probiotic intake could prevent up to 60% more NDD cases, with a 78% potential increase if childhood probiotic intake were consumed by all populations, particularly in the gross motor, fine motor and personal-social domains. These findings highlight the importance of early-life dietary supplements in NDD prevention, particularly the enhanced protective effect of childhood probiotic intake in combination with prenatal multivitamin supplementation. Despite the promising results, future prospective studies with detailed data are needed to confirm this enhanced effect of childhood probiotic intake and their underlying mechanisms.

## Data Availability

The raw data supporting the conclusions of this article will be made available by the authors without undue reservation.
