# Supplementary material for: The effects of early childhood probiotic intake on the association between prenatal micronutrient supplementation and neurobehavioral development in preschool children: a four-way decomposition analysis
Source: Front Nutr. 2025 May 21;12:1614820. doi: 10.3389/fnut.2025.1614820 (PMC12133521; doi:10.3389/fnut.2025.1614820)
Supplement: Supplementary file 1 [file Data_Sheet_1.ZIP › Stable 1_definition&code.docx]

**Supplementary Table 1 Definitions and coding used in the questionnaire of 2022 children survey.**

| **Variable** | **Question** | **Values** |
| --- | --- | --- |
| Child’s age | What is the child's birth date? | - |
| Birth weight | What was the birth weight of child? (kg) | - |
| Maternal/ paternal age of conception | What is the mother's birth date?  What is the father's birth date? | Mother's age - child's age  Father's age - child's age |
| Child’s sex | What is the sex of the child? | 1= Male  2= Female |
| Birth season | What is the child's birth date? | 1= Spring  2= Summer  3= Autumn  4= Winter |
| Residence type | What is the child's residence type? | 1 = Shenzhen residents  2 = Non-Shenzhen residents (including temporary and floating residents) |
| Separation from mother/father | Was the child ever separated from mother/father for more than 6 months? | 1= No  2= Yes |
| Marital status | What is the mother's marital status? | 1= Not married (including single, divorced and widowed)  2= Married (including married and remarried) |
| Mother/father education | What is the mother's/father’s education level? | 1= Less than high school  2= High school and higher |
| Household income | What is your total monthly household income? | 1= <RMB 20,000  2= ≥RMB 20,000 |
| Pregnancy mode | What was the pregnancy mode for this child? | 1= Natural pregnancy  2= Medically assisted pregnancy |
| Threatened abortion (TA) | Did the mother have a threatened abortion during the pregnancy? | 1= No  2= Yes |
| Intrauterine growth retardation (IUGR) | Was the child diagnosed with intrauterine growth retardation? | 1= No  2= Yes |
| Congenital diseases | Was the child diagnosed with congenital diseases? | 1= No  2= Yes |
| Pregestational diabetes mellitus (PGDM) | Had the mother ever been diagnosed with diabetes mellitus before pregnancy with this child? | 1= No  2= Yes |
| Pregestational hypertension(PGH) | Had the mother ever been diagnosed with hypertension before pregnancy with this child? | 1= No  2= Yes |
| Pregestational mental disease(PGMD) | Had the mother ever been diagnosed with mental disease before pregnancy with this child? | 1= No  2= Yes |
| Polycystic ovarian syndrome (POS) | Had the mother ever been diagnosed with polycystic ovarian syndrome before pregnancy with this child? | 1= No  2= Yes |
| Gestational hypertension (GH) | Was the mother diagnosed with gestational hypertension during the pregnancy with this child? | 1= No  2= Yes |
| Pre-eclampsia (PE) | Was the mother diagnosed with pre-eclampsia during the pregnancy with this child? | 1= No  2= Yes |
| Gestational diabetes mellitus (GDM) | Was the mother diagnosed with gestational diabetes mellitus during the pregnancy with this child? | 1= No  2= Yes |
| Pregnancy Chinese medicine intake | Did the mother take Chinese medicine during the pregnancy? | 1= No  2= Yes |
| Pregnancy preservation | Did the mother take pregnancy preservation during the pregnancy? | 1= No  2= Yes |
| Pregnancy seminar participation | Did the mother participate in pregnancy seminars during the pregnancy? | 1= No  2= Yes |
| Pre-pregnancy BMI | What were the mother's height (m) and weight (kg) before this pregnancy? | 1= BMI <18.5 kg/m^2^  2= BMI 18.5-23.9 kg/m^2^  3= BMI ≥24 kg/m^2^ |
| Parity | How many times had the mother given birth before this child? | 1= Nulliparous  2= Multiparous |
| Multiple pregnancy | How many fetuses was the mother carrying? | 1 = Single pregnancy  2 = Multiple pregnancy |
| Delivery mode | What was the mother's delivery model for this child? | 1= Natural birth  2= Cesarean  3= Vaginal surgery for delivery |
| Preterm birth (PTB) | What was the gestational age of child? (Week) | 1 = No (≥37 weeks)  2 = Yes (<37 weeks) |
| Feeding pattern | What was the child's feeding pattern in early childhood? | 1= Breastfeeding  2= Formula feeding  3= Mixed feeding |
| Child’s nutritional condition | What was the child's nutritional status in early childhood? | 1= Bad (very bad/ bad)  2= Good (general/good/very good) |
| Child’s health condition | What was the child's health status in early childhood? | 1= Bad (very bad/ bad)  2= Good (general/good/very good) |
| Mother/father smoking | Does the mother/father smoke? | 1= No  2= Yes |
| Mother/father drinking | Does the mother/father drink alcohol? | 1= No  2= Yes |
| Parental depression | PHQ-9 | 1= No (Score >4)  2= Yes (Score ≤4) |
| Family function | Family APGAR scale | 1= Normal (Score ≥7)  2= Dysfunction (Score <7) |
