# Supplementary material for: The effects of early childhood probiotic intake on the association between prenatal micronutrient supplementation and neurobehavioral development in preschool children: a four-way decomposition analysis
Source: Front Nutr. 2025 May 21;12:1614820. doi: 10.3389/fnut.2025.1614820 (PMC12133521; doi:10.3389/fnut.2025.1614820)
Supplement: Supplementary file 1 [file Data_Sheet_1.ZIP › Stable 3 Associations between micronutrient & NND.docx]

**Supplementary Table 3 Association between prenatal micronutrient supplementation and NDD in the crude, adjusted and full-inclusion model.**

| Variables | NDD | | | | | | |
| --- | --- | --- | --- | --- | --- | --- | --- |
|  |  | Crude Model | | Adjusted Model^a^ | | Full-inclusion Model^b^ | |
|  | *N*(%) | OR(95%CI) | *p*-value | OR(95%CI) | *p-value* | OR(95%CI) | *p-value* |
| Calcium |  |  |  |  |  |  |  |
| No | 495 (13.1) | 1.00 |  | 1.00 |  | 1.00 |  |
| Yes | 1,337 (11.3) | 0.84 (0.76,0.94) | **0.003** | 0.92 (0.82,1.03) | 0.16 | 0.93 (0.80,1.07) | 0.32 |
| Folic acid |  |  |  |  |  |  |  |
| No | 249 (13.5) | 1.00 |  | 1.00 |  | 1.00 |  |
| Yes | 1,583 (11.5) | 0.83 (0.72,0.96) | **0.01** | 0.90 (0.78,1.05) | 0.18 | 0.93 (0.78,1.11) | 0.42 |
| Iron |  |  |  |  |  |  |  |
| No | 1,023 (12.1) | 1.00 |  | 1.00 |  | 1.00 |  |
| Yes | 809 (11.3) | 0.92 (0.84,1.02) | 0.10 | 1.05 (0.95,1.17) | 0.30 | 1.14 (1.01,1.28) | 0.03 |
| Multivitamin |  |  |  |  |  |  |  |
| No | 1,141 (13.1) | 1.00 |  | 1.00 |  | 1.00 |  |
| Yes | 691 (9.9) | 0.73 (0.66,0.81) | **<0.001** | 0.86 (0.78,0.96) | **0.007** | 0.85 (0.75,0.95) | **0.006** |

^a^ Adjusted Model: Adjusted for child’s demographic characteristics, maternal demographic characteristics, pregnancy and perinatal characteristics and childhood family environment.

^b^ Full-inclusion Model: Included all micronutrients in the model while adjusting for child’s demographic characteristics, maternal demographic characteristics, pregnancy and perinatal characteristics and childhood family environment.
